# Role of microRNAs in the treatment of type 2 diabetes mellitus with Roux-en-Y gastric bypass

**DOI:** 10.1590/1414-431X20175817

**Published:** 2017-03-02

**Authors:** Z. Zhu, J. Yin, D.C. Li, Z.Q. Mao

**Affiliations:** The First Affiliated Hospital of Soochow University, Suzhou, Jiangsu Province, China

**Keywords:** Expression profile, Methylation, MicroRNA, Roux-en-Y gastric bypass, Type 2 diabetes mellitus

## Abstract

The aim of this study was to investigate the effect of Roux-en-Y gastric bypass (RYGB) on the peripheral blood microRNAs (miRNAs) of patients with type 2 diabetes mellitus (T2DM). miRNAs are small 20- to 22-nucleotide (nt) noncoding RNAs. They constitute a novel class of gene regulators that negatively regulate gene expression at the post-transcriptional level. miRNAs play an important role in several biological processes. Twelve patients with T2DM who were scheduled to undergo laparoscopic RYGB surgery were separated into two groups, using a body mass index of 30 kg/m^2^ as a cut-off point. Venous blood was collected before operation and 12 months after operation. A significant change was observed in the peripheral blood miRNA expression profile of both groups after RYGB surgery compared with those before operation. The expression levels of hsa-miR-29a-3p, hsa-miR-122-5p, hsa-miR-124-3p, and hsa-miR-320a were downregulated. The methylation state of the CpG sites within an approximately 400-bp genomic DNA fragment of each of the four miRNA genes, including about 200 bp upstream and 100 bp downstream of the pre-miRNA, did not vary after RYGB surgery. With remission of T2DM in both groups, RYGB could modulate the expression level of many peripheral blood miRNAs associated with lipid metabolism, insulin secretion, beta-cell function, and insulin resistance. The expression level of peripheral blood diabetes-related miRNA varied in patients with T2DM after receiving RYGB surgery, laying a strong foundation for future studies on this subject. The molecular mechanisms underlying RYGB surgery that can cause aberrant expression of miRNA remains to be determined.

## Introduction

Type 2 diabetes mellitus (T2DM) is a long-term metabolic disorder that is characterized by high blood sugar, insulin resistance, and relative lack of insulin. The prevalence of T2DM has increased markedly since 1960 in parallel with obesity. As of 2013, approximately 368 million people were diagnosed with the disease compared with around 30 million in 1985 (1,2). T2DM is associated with a 10-year shorter life expectancy. Roux-en-Y gastric bypass (RYGB) is prescribed to treat morbid obesity (defined as a body mass index (BMI) greater than 40), T2DM, hypertension, sleep apnea, and other comorbid conditions. Bariatric surgery is the term encompassing all of the surgical treatments for morbid obesity; gastric bypass is only one class of such operations. The resulting weight loss, typically dramatic, markedly reduces comorbidities. The long-term mortality rate of gastric bypass patients has been shown to be reduced by up to 40% (3,4). The exact mechanism, however, remains unclear.

MicroRNAs (miRNAs) are small 20- to 22-nucleotide (nt) noncoding RNAs. They constitute an abundant class of gene regulators that negatively regulate gene expression at the post-transcriptional level. They bind with the 3′ untranslated regions of their mRNA targets and repress target-gene expression by mRNA degradation or translational repression. Several miRNAs have been implicated in controlling both insulin signaling and glucose metabolism at multiple levels (5). They may also play an important role in the effects of RYGB. The precise mechanisms regulating miRNA expression are largely unknown, but studies have suggested several, including genetic and epigenetic alterations (6,7). Current research in this area, however, is lacking. The primary aim of this study is to investigate the effect of RYGB on the expression level of serum miRNAs of patients with T2DM and explore the probable mechanism.

## Material and Methods

### Ethical approval

This study received approval from the Institutional Review Board of the First Affiliated Hospital of Soochow University (Suzhou, China). All procedures involving human participants were in accordance with the 1964 Helsinki declaration and its later amendments or comparable ethical standards. (Ethical approval No. 2010264088). Informed consent was obtained from all individual participants included in the study.

### Subjects

The study included 12 patients with T2DM, BMI ≥27.5 kg/m^2^, age ≤65 years, and course of disease ≤15 years; the C-peptide level was ≥2 times higher in the oral glucose tolerance test (OGTT), which was the operation criterion. The control group comprised three morbidly obese patients without T2DM, whose BMI was >40 kg/m^2^. The 12 patients with T2DM were divided into two groups: high-BMI T2DM group (n=10) and low-BMI T2DM group (n=2), using BMI of 30 kg/m^2^ as a cut-off point.

### Surgery and samples

All patients underwent laparoscopic RYGB surgery. The size of the gastric pouch was about 30 mL, and the biliary and Roux limbs were both 100 cm long. Venous blood samples were collected prior to surgery and 1 year postoperatively, and stored in EDTA-coated vacuum tubes. RNase-free protocols were followed throughout the procedures. Peripheral blood mononuclear cells (PBMCs) were isolated from EDTA tubes using red blood cell lysis buffer (8.3 g NH_4_Cl, 1.0 g KHCO_3_, 1.8 mL 5% EDTA in distilled H_2_O). Total RNA was isolated from PBMCs using Trizol (Invitrogen, China) according to manufacturer instructions and eluted in 30 μL of nuclease-free water (Takara Bio Inc., Japan).

### Microarray analysis

The RNA samples of one patient in the high-BMI T2DM group and one from a healthy volunteer (as a control) were sent to Super Biotek Corporation in Shanghai for determining the expression profile of the serum miRNAs. All samples were preserved in dry ice, and passed quality-control testing. Each sample was performed in triplicate. Bioinformatics analyses were performed of the microarray data, miRBase miRNA target-gene data (http://microrna.sanger.ac.uk/targets/v5/), and relative articles on metabolic diseases (8 [Bibr B09]–[Bibr B11] 12). Then, the following four miRNAs that might underlie the treatment effect of bariatric surgery were chosen for further quantitative real-time polymerase chain reaction (qRT-PCR) analysis: hsa-miR-29a-3p, hsa-miR-122-5p, hsa-miR-124-3p, and hsa-miR-320a.

### Quantitative RT-PCR

qRT-PCR was used to determine the expression level of hsa-miR-29a-3p, hsa-miR-122-5p, hsa-miR-124-3p, and hsa-miR-320a in all RNA samples. cDNA was synthesized from 10 μg of total RNA, using a Mir-X miRNA First-Strand Synthesis Kit (Clontech Laboratories, USA). qRT-PCR was performed with SYBR Premix Ex Taq (TaKaRa) following the manufacturer's protocol. Normalization was performed with a small nuclear RNA U6 (Mir-X miRNA First-Strand Synthesis Kit, Clontech Laboratories). The miRNA-specific primer sequences and the PCR reaction conditions are listed in Table 1. All real-time reactions, including non-template controls, were run using the LightCycler 480 System (Roche Diagnostics Ltd., Switzerland) and performed in triplicate. Relative quantities of miRNA were calculated using the ΔΔCT method after normalization with reference to the expression of RNA U6 as endogenous control. The difference in the expression level of miRNA between a preoperative sample and a postoperative sample was compared using the Student *t*-test, with P<0.05 considered to be statistically significant.

**Table:**
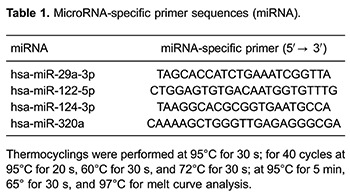

### Methylation detection

Both preoperative and postoperative genomic DNAs of one patient in the high-BMI T2DM group were extracted from PBMCs, using an AxyPrep Multisource Genomic DNA Miniprep Kit (Axygen, USA) following the manufacturer's protocol. The DNA specimens were sent to Super Biotek Corporation (China) for bisulfite sequencing PCR (BSP). An approximately 400-bp genomic DNA fragment of each of the four miRNA genes, including about 200 bp upstream and about 100 bp downstream of the pre-miRNA, was amplified by PCR. This genomic DNA fragment contained putative core promoter region of a miRNA gene (13). The BSP of each miRNA gene was repeated five times.

## Results

### Clinical follow-up data

One year after RYGB, the OGTT, blood glucose, BMI, and glycosylated hemoglobin A1c (HbA1c) were all significantly improved (Table 2). All patients stopped insulin treatment 1 year postoperatively. It was also found that the treatment effect of RYGB was more profound in the high-BMI group and T2DM was remitted immediately after surgery, before weight loss.

**Table:**
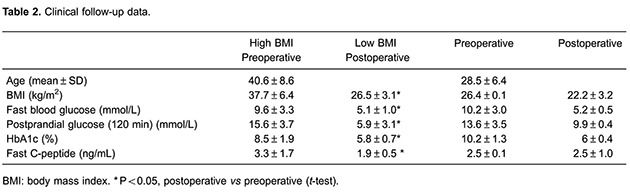

### Changes in the expression levels of serum miRNAs after RYGB

The microarray data showed 70 miRNAs upregulated and 74 miRNAs downregulated after RYGB in 1 patient of the high-BMI group. Among them, hsa-miR-29a-3p, hsa-miR-122-5p, hsa-miR-124-3p, and hsa-miR-320a were chosen for further analysis. qRT-PCR was used to determine the expression level of these four miRNAs in all RNA samples.

**Figure 1:**
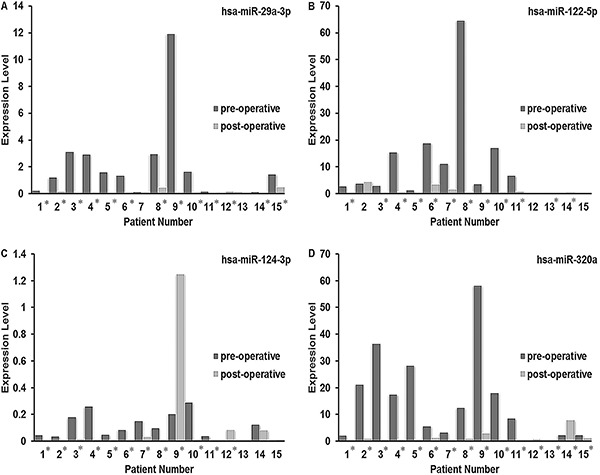
Quantitative RT-PCR for hsa-miR-29a-3p (*A*), hsa-miR-122-5p (*B*), hsa-miR-124-3p (*C*), and hsa-miR-320a (*D*). Relative quantities of miRNA were normalized with reference to the expression of RNA U6 as endogenous control. High-BMI T2DM group: patients No. 1-10; low-BMI T2DM group: patients No. 11 and 12; morbid obesity group: patients No. 13-15. Data are reported as ΔΔCT value. *P<0.05 postoperative *vs* preoperative (*t*-test).

For all groups, hsa-miR-29a-3p was downregulated in 10 patients with T2DM and 2 morbidly obese patients, and upregulated in 1 patient with T2DM postoperatively compared with the preoperative period. hsa-miR-122-5p was downregulated in 10 patients with T2DM and upregulated in 2 morbidly obese patients. hsa-miR-124-3p was downregulated in 10 patients with T2DM and upregulated in 2 patients with T2DM. hsa-miR-320a was downregulated in 11 patients with T2DM and 1 morbidly obese patient, and upregulated in 1 patient with T2DM and 2 morbidly obese patients (Figure 1). The expression level of these four miRNAs was much higher in the two T2DM groups than in the morbid obesity group in the preoperative period. In both T2DM groups, hsa-miR-122-5p and hsa-miR-320a showed notable downregulation. These data suggested that miRNA might play an important role in the treatment of T2DM with RYGB.

### Changes in DNA methylation levels around miRNA genes after RYGB

BSP was performed to determine whether the methylation level of about 400-bp genomic DNA fragment of each pre-miRNA and about 200 bp upstream and about 100 bp downstream flanking sequences at each end of the precursor changed after RYGB. The results revealed 4 methylated CpG sites in the DNA fragment around hsa-miR-29a-3p, 5 methylated CpG sites in the DNA fragment around hsa-miR-122-5p, 2 methylated CpG sites and 10 unmethylated CpG sites in the DNA fragment around hsa-miR-124-3p, and 1 methylated CpG site and 43 unmethylated CpG sites in the DNA fragment around hsa-miR-320a preoperatively. None of the methylation levels of the DNA fragment around miRNA genes changed postoperatively. These results indicated that the change in DNA methylation level might not represent the main mechanism underlying modulation of the miRNA gene transcription.

## Discussion

The growing incidence of morbid obesity and T2DM globally is widely recognized as one of the most challenging contemporary threats to public health (14). Uncontrolled T2DM leads to macrovascular and microvascular complications, including myocardial infarction, stroke, blindness, neuropathy, and renal failure in many patients. Despite improvements in pharmacotherapy, fewer than 50% of patients with moderate-to-severe T2DM actually achieve and maintain therapeutic thresholds, particularly for glycemic control (15). Current studies have suggested that bariatric surgery, including RYGB, can rapidly improve glycemic control and cardiovascular risk factors in severely obese patients with T2DM (16,17). RYGB, as a commonly used bariatric surgical procedure, can effectively reduce body weight and treat T2DM, with a cure rate as high as 80% (18). The clinical follow-up data in this study also fully validated this rate (Table 2). However, currently many theories exist about the mechanism by which bariatric surgery achieves these results, which require further investigation.

MiRNAs are members of a rapidly growing class of small noncoding RNAs, 19-23 nt in length, known to contribute to the regulation of gene expression in plants and animals. miRNAs act on target mRNAs in a sequence-specific manner to either promote their cleavage and degradation or reduce their translational efficiency. Several miRNAs have recently been implicated in controlling both insulin signaling and glucose metabolism at multiple levels (7). This study, using high-throughput screening of miRNA microarray first and then quantitative RT-PCR, attempted to explore the impact of RYGB surgery on peripheral blood miRNA expression profiles of patients with T2DM. One patient in the high-BMI group was screened by miRNA microarray assay. A total of 70 miRNAs were upregulated and 74 miRNAs were downregulated after RYGB. Four miRNAs were proven to be related to the development and progression of T2DM, including hsa-miR-29a-3p, hsa-miR-122-5p, hsa-miR-124-3p, and hsa-miR-320a (8–10).

One study showed that miR-29a was a mediator of glucose-induced beta-cell dysfunction, and glucose-induced upregulation of miR-29a in beta cells could be implicated in progression from impaired glucose tolerance to T2DM (19). In this study, hsa-miR-29a-3p was downregulated in 10 patients with T2DM (9 high-BMI and 1 low-BMI) and 2 morbidly obese patients after RYGB surgery. These data suggested that RYGB might remit glucose-induced beta-cell dysfunction and stop the onset of T2DM. miR-122 was reported as a key regulator of cholesterol and fatty acid metabolism in the adult liver (20). miR-122 inhibition resulted in reduced plasma cholesterol levels, increased hepatic fatty acid oxidation, and a decrease in hepatic fatty acid and cholesterol synthesis. hsa-miR-122-5p was found to be downregulated in 10 patients with T2DM (9 high-BMI and 1 low-BMI) and upregulated in 2 morbidly obese patients postoperatively. However, the expression levels of hsa-miR-122-5p were extremely lower in the morbidly obese patients compared with the patients with T2DM (Figure 1). This indicated that RYGB might reduce the expression level of overexpressed hsa-miR-122-5p and, subsequently, of circulating total cholesterol and triglycerides. Interestingly, hsa-miR-122-5p was overexpressed in patients with preoperative T2DM compared with morbidly obese patients, which meant that hsa-miR-122-5p might play a role in the development and progression of T2DM. Further studies are needed to verify this theory. It has been reported that overexpression of miR-124a leads to a reduction in glucose-induced insulin secretion (21). hsa-miR-124-3p was downregulated in 10 patients with T2DM (9 high-BMI and 1 low-BMI) and upregulated in 2 patients with T2DM (1 high-BMI and 1 low-BMI) after surgery. This implied that RYGB might improve glucose-induced insulin exocytosis in most patients with T2DM. Several studies reported that miR-320 might regulate insulin sensitivity of adipocytes (22,23). Anti-miR-320 oligonucleotide was found to regulate insulin resistance in adipocytes by improving insulin-PI3-K signaling pathways. Also, the beneficial effects of anti-miR-320 oligo were observed only in insulin resistance (IR) adipocytes and not in normal adipocytes. In this study, hsa-miR-320a was found to be downregulated in 11 patients with T2DM and 1 morbidly obese patient 1 year after surgery. Meanwhile, hsa-miR-320a was upregulated in one patient with T2DM of the low-BMI group and two morbidly obese patients. The expression levels of hsa-miR-320a were notably lower in these three patients than in other patients with T2DM in the preoperative period (Figure 1), indicating that IR might not exist in these patients. These results corresponded to the aforementioned studies. Therefore, RYGB might cure the IR of adipocytes in patients with T2DM. These results might indicate that peripheral blood miRNA could be an important factor for T2DM improvement after RYGB independently of BMI.

Given that obesity is a major risk factor for IR and a known trigger for the onset of T2DM (24), weight loss significantly alleviates IR, which is consistent with the present clinical data. The result of the peripheral blood diabetes-related miRNA regulation postoperatively was also consistent with reduction of IR, increasing glucose-induced insulin secretion, and protection of beta-cell function. However, in the low-BMI group, as the magnitude of weight loss due to the RYGB was relatively small, the effect of improving IR was relatively weak. Even so, the differences in the type and magnitude of the peripheral blood diabetes-related miRNA compared with the preoperative period were still significant in this study, indicating that miRNA continued to play a role in regulating insulin secretion, improving IR, and protecting beta-cell function. Hence, it was observed that after RYGB, the expression level of diabetes-related miRNA in patients with T2DM changed to protect beta-cell function, promote glucose-induced insulin secretion, and reduce IR. This was not consistent with the degree of improvement in obesity symptoms, indicating that RYGB might prevent and remit T2DM directly before weight loss, through regulating the expression level of diabetes-related miRNA. This suggested that miRNA might have a unique role in treating T2DM by RYGB.

With the process of RYGB surgery for treating T2DM, multiple direct or indirect factors, such as effects in energy metabolism and hormones, changes in the gastrointestinal tract, weight loss, and so forth, can influence the peripheral blood diabetes-related miRNA. These factors can regulate the downstream insulin signaling pathway. Also, factors related to the etiology of T2DM might play a role. This kind of change was not entirely determined by the reduction of weight and body fat content.

In summary, this study presented evidence that the expression level of peripheral blood diabetes-related miRNA varied in patients with T2DM after receiving RYGB surgery, laying a strong foundation for future studies on this subject. The molecular mechanism underlying RYGB surgery that can cause aberrant expression of miRNA remains to be determined.
